# Deciphering tumour microenvironment and elucidating the origin of cancer cells in ovarian clear cell carcinoma

**DOI:** 10.1101/2024.08.06.606821

**Published:** 2024-08-08

**Authors:** Uma S Kamaraj, Pradeep Gautam, Terence Cheng, Tham Su Chin, Sun Kuie Tay, Tew Hong Ho, Ravichandran Nadarajah, Ronald Chin Hong Goh, Shing Lih Wong, Sangeeta Mantoo, Inny Busmanis, Hu Li, Minh TN Le, Qi-Jing Li, Elaine Hsuen Lim, Yuin-Han Loh

**Affiliations:** 2 Institute of Molecular and Cell Biology (IMCB), Agency for Science, Technology and Research (A*STAR), 61 Biopolis Drive, Proteos, Singapore 138673, Republic of Singapore; 3 Department of Obstetrics & Gynaecology, Singapore General Hospital, Outram Road, Singapore 169608; 4 Department of Anatomical Pathology, Singapore General Hospital, Academia, College Road, Singapore 169856; 5 Center for Individualized Medicine, Department of Molecular Pharmacology and Experimental Therapeutics, Mayo Clinic, Rochester, MN 55905, USA; 6 Yong Loo Lin School of Medicine, National University of Singapore, Singapore; 7 Division of Medical Oncology, National Cancer Centre Singapore, 30 Hospital Boulevard, Singapore 168583; 8 Department of Physiology, NUS Yong Loo Lin School of Medicine, 2 Medical Drive, MD9, Singapore, Singapore; 9 Department of Biological Sciences, National University of Singapore, Singapore, Singapore; 10 NUS Graduate School’s Integrative Sciences and Engineering Programme, National University of Singapore, 28 Medical Drive, Singapore, Singapore.

## Abstract

Ovarian clear cell carcinoma (CCC) has an East Asian preponderance. It is associated with endometriosis, a benign condition where endometrial (inner lining of the uterus) tissue is found outside the uterus and on the peritoneal surface, in the abdominal or pelvic space. CCC is relatively more resistant to conventional chemotherapy compared to other ovarian cancer subtypes and is associated with a poorer prognosis. In this study, we recruited and obtained tumour tissues from seven patients across the four stages of CCC. The tumour and the tumour microenvironment (TME) from 7 CCC patients spanning clinical stages 1–4 were transcriptionally profiled using high-resolution scRNA-seq to gain insight into CCC’s biological mechanisms. Firstly, we built a scRNA-seq resource for the CCC tumour microenvironment (TME). Secondly, we identified the different cell type proportions and found high levels of immune infiltration in CCC. Thirdly, since CCC is associated with endometriosis, we compared CCC with two publicly available endometriosis scRNA-seq datasets. The CCC malignant cells showed similarities with glandular secretory and ciliated epithelial cells found in endometriosis. Finally, we determined the differences in cell-cell communication between various cell types present in CCC TME and endometriosis conditions to gain insights into the transformations in CCC.

## Introduction

Ovarian cancer (OC) is a lethal gynaecological cancer affecting women globally and is the 7^th^ most common cause of female cancer-associated death^1,2^. The World Health Organisation’s (WHO) International Agency for Research on Cancer (IARC) estimates ovarian cancer to increase in incidence and mortality in the next two decades^3^. The high mortality rates associated with OC are primarily due to the advanced disease stage at presentation and the lack of effective screening tools. It is categorised into four subtypes: serous, endometroid, clear cell, and mucinous^4^. Ovarian clear cell carcinoma (CCC) exhibits different prevalence rates across various regions, representing 5–10% of cases in North America but ranging from 13–25% in East Asia^5,6^. The greater East Asian preponderance is mirrored by the higher incidence of endometriosis in East Asia^7–10^, CCC being the subtype more highly correlated with endometriosis^11–16^.

Endometriosis is an inflammatory, non-malignant lesion affecting 10% of women of reproductive age worldwide^17^, causing pelvic pain and infertility. Sampson’s theory of retrograde menstruation^18^ postulated that endometrial cells moved from the inner lining of the uterus, through the Fallopian tubes, and into the abdominopelvic cavity. Women with endometriosis are three times more likely to develop CCC^16^. The pattern of distribution of endometriotic lesions in the abdominal or pelvic space is similar to ovarian cancer metastasis. Oncogenic mutations in *ARID1A*, *PIK3CA*, *KRAS* and *PPP2R1A*^19^ have been described in endometriotic tissue. *PIK3CA* mutation has been described as an early event in CCC^20^. Altogether, these data suggest that endometriosis is less-than-benign, prompting an enduring clinical question as to whether endometriosis is a precursor or facilitator of CCC.

Our study compares scRNA-seq data on CCC with endometriosis to identify the precursor cells for CCC. A recent study identified two expression subtypes in CCC: epithelial-like (associated with earlier stages) and mesenchymal-like (associated with later stages)^21^. Recently, single-cell RNA sequencing (scRNA-seq) technology^22,23^ has been applied to decipher normal and tumour conditions. Two recent cohort studies have used scRNA-seq to describe endometriosis^24,25^. However, there is currently a lack of high-resolution scRNA-seq data on CCC. To fill this gap, we present scRNA-seq profiling of CCC patients in our cohort, which includes various clinical stages of CCC. We used previously published studies on endometriosis to understand the differences and associations between CCC and endometriosis and, as well as to elucidate the origin of ovarian CCC cells. Analysing the differential genes expressed in heterogenous cell types present in CCC compared across the controls, we sought to acquire molecular insights that might clarify the role of endometriosis in CCC. Further, we provide insights into the critical TME cellular communications, thereby identifying molecules and pathways potentially targetable by existing drugs or further therapeutic development.

## Results

### Cellular composition in CCC tumour microenvironment

**Figure 1:**
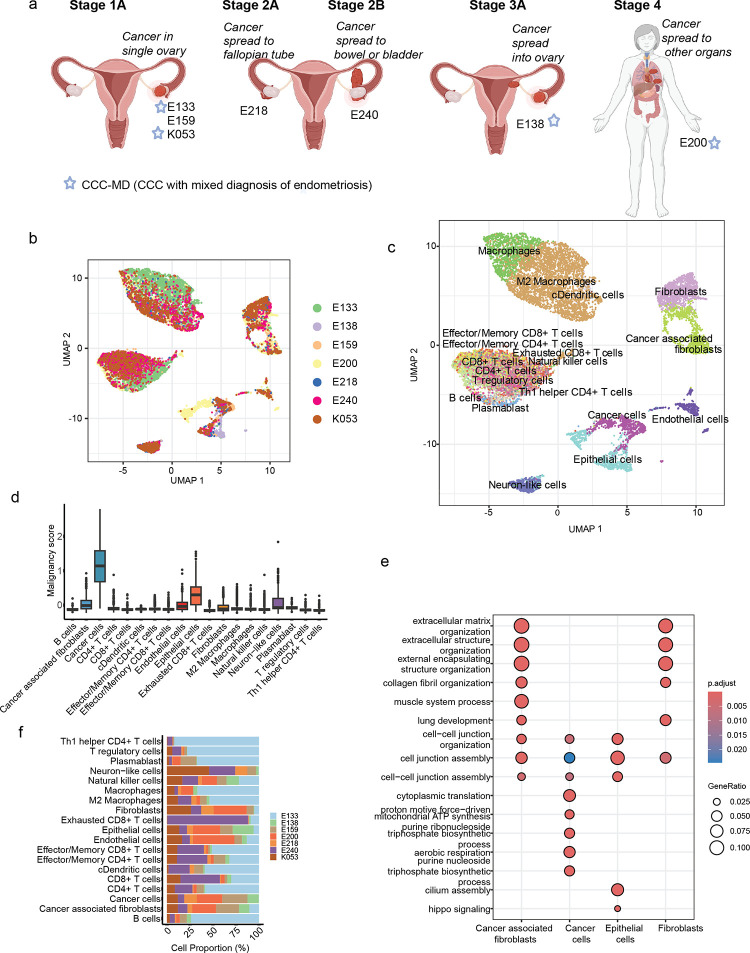
Overview of CCC samples used in this study and cellular composition of tumour microenvironment. a) The schematic shows the overview of the tumour samples from seven patients across various clinical stages of CCC. CCC refers to ovarian clear cell carcinoma, and CCC-MD refers to CCC with a mixed diagnosis of endometriosis. The schematic was created in BioRender.com. b) The integrated scRNA-seq data of the CCC tumour samples from patients were visualised using UMAP dimension reduction. c) The cell types identified in the samples were visualised using UMAP. d) The boxplot shows the malignancy score based on cancer-associated genes across all the cell types in CCC. e) The dot plot shows the gene ontology enrichment in selected epithelial cells, cancer cells, fibroblasts and cancer-associated fibroblasts across all the samples. f) The percentage of different cell types in the tumours of each of the patients.

The samples of surgically resected ovarian CCC tissue from seven patients of different disease stages, including FIGO stages 1A, 2A, 2B, 3A and 4 were collected (Figure 1a, Table S1). The H&E staining of the CCC samples showed that these samples have abnormal morphologies with multilobed nuclei (Figure S1a). Of these, three patients were in stage 1 with cancer in a single ovary, two patients were in stage 2 where cancer has spread to the bladder or bowel, one in stage 3 where cancer spread into the ovary and one in stage 4 where cancer has metastasised and spread to other organs. Additionally, three of these seven patients were diagnosed with endometriosis along with CCC and are referred to as CCC-MD (Figure 1a). To understand the cellular composition within the tumour sample, we performed high-resolution scRNA-seq on the seven patient tissues and integrated the cells across the patients (Figure 1b). Following quality control, we obtained 13,543 cells across these CCC samples to dissect the tumour microenvironment (TME) (Figure S1b-e).

To determine the cell types in the tumour samples, we used scATOMIC^26^, a model trained with the transcriptional profiles of the cell types annotated from publicly available scRNA-seq datasets on TMEs across 19 common cancers, including ovarian cancer. Using scATOMIC and manual curation, we annotated the different cell types in the CCC TME (Figure 1c). We observed 19 cell types in the CCC, including epithelial cells, endothelial cells, fibroblasts, neurons, and immune cells. The cells annotated as cancer cells have higher expressions of malignancy markers obtained from the CancerSCEM^27^ database (Figure 1d). Consistent with the previous studies, cells annotated as B cells express *CD79A*, *CD19*, and *MS4A1*^28^, exhausted T cells express *PDCD1*^29^, Treg cells express *FOXP3*^30^. Macrophages in CCC samples were separated into two groups, M2 macrophages and macrophages based on the high expression of *CD86* and low expression of *CCL5*^31^. Further, cancer-associated fibroblasts were annotated based on the expression of *ACTA2* and *TAGLN*^32^ (Figure S1f). The epithelial and cancer cells showed higher *KRT8*, *KRT18*, *KRT19* and *ANXA4* expression^27^.

Subsequently, differential gene ontology biological processes indicated that the cancer cells had enrichment for cell division and proliferation-related terms compared to the epithelial cells, and the cancer-associated fibroblasts showed enrichment of cell-cell assembly-related gene ontology biological processes (Figure 1e, S1g). *ARID1A* and *PIK3CA* are well-known oncogenes associated with CCC^33^, and here, we also found a higher mutation rate at these genes across the samples (Figure S2a-b). Based on cell population, a significantly higher proportion of immune cells were present in all the CCC tumours compared to epithelial or fibroblasts, indicating an immune infiltration at the tumour site (Figure 1f).

### Immune infiltration and cellular communication in TME

**Figure 2:**
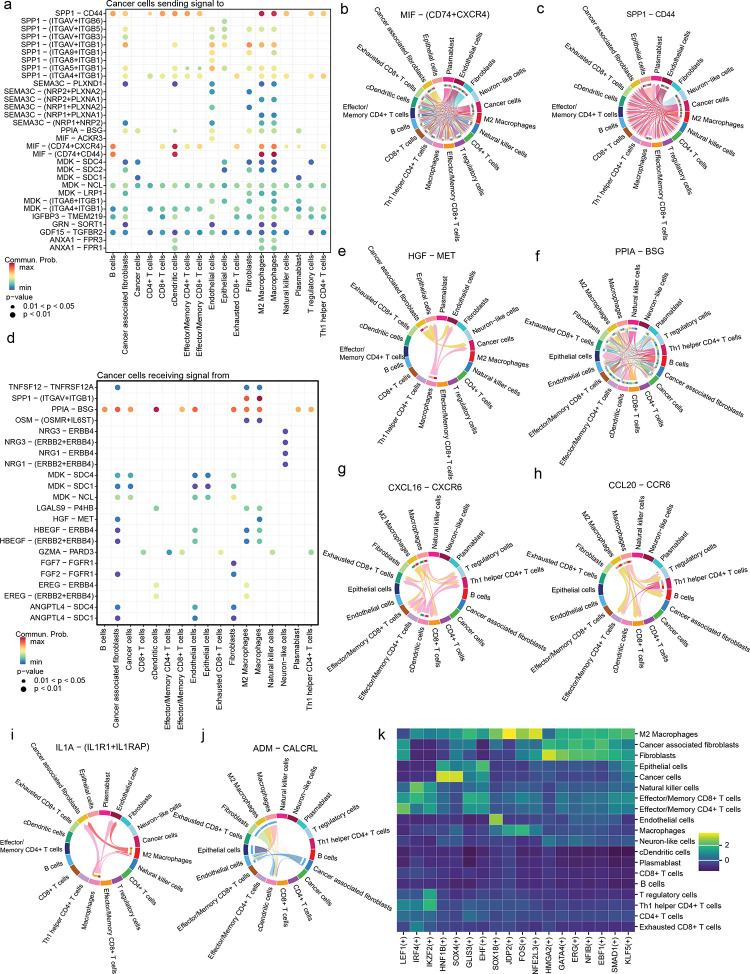
Cell-cell communication signalling in the tumour microenvironment: a) The dot plot shows the significant ligands the cancer cells produce targeting the receptors in the ligand-receptor (L-R) signalling. The chord plots show the ligand and receptor interaction among the cell types in the TME for the following selected signalling pathways: b) MIF pathway with MIF ligand and *CD74*-*CXCR4* receptor, c) SPP1 pathway with *SPP1* ligand and *CD44* receptor. The colour of the connections between cell types represents the ligands expressed from the cell type and the arrow points to the receptor of the cell types. d) The dot plot shows the significant receptors that are active in the cancer cells for the ligands produced by other cell types in the TME. The chord plots show the ligand and receptor interaction among the cell types in the TME for the following selected signalling pathways: e) HGF pathway with HGF ligand and MET receptor, f) CypA pathway with *PPIA* ligand and *BSG* receptor, g) CXCL pathway with *CXCL16* ligand and *CXCR6* receptor, h) CCL pathway with *CCL20* ligand and *CCR7* receptor, i) IL1 pathway with *IL1A* ligand and *IL1R1*-*IL1RAP* receptor and j) CALCR pathway with *ADM* ligand and *CALCRL* receptor. k) The heatmap shows the enrichment of TFs regulons with regulon specificity scores in the cell types found in TME.

Henceforth, we sought to decipher the cell-cell communications within the TME of the collected patient tumour samples. The top signalling pathways active in the TME across all samples were pathways such as MIF, TGFb, PARs, SPP1 and CXCL (Figure S2c). The cancer cells in CCC predominately secreted ligands for pathways such as MIF and SPP1 (Figure 2a). The cancer cells and cancer-associated fibroblasts produced the MIF ligands, signalling to the immune cells such as macrophages and T cells (Figure 2b). Interestingly, the surrounding normal epithelial cells in CCC are not involved in *MIF*-*CD74* signalling and are cancer-specific. *MIF* cytokine is known to have a pro-tumour role with functions covering several hallmarks of cancer, including resisting cell death, inducing angiogenesis, promoting genome instability, tumour proliferation and acting as an immunosuppressor^34^. Similarly, the SPP1 pathway showed significant signalling interaction between cancer and macrophages, and *SPP1* is a poor prognostic marker for clear cell carcinoma^35,36^. Cancer-associated fibroblasts signal to the cancer cells for tumour growth and angiogenesis via pathways such as HGF, ANGPTL4, HBEGF and FGF (Figure 2d). Dysregulation of the *HGF*-*MET* axis is well-known in tumorigenesis and invasion^37^, and here in CCC, cancer-associated fibroblasts and macrophages signal the cancer cells (Figure 2e). Similarly, *PPIA* and *BSG* co-expression is significantly associated with poor prognosis in tumours^38^, and in CCC the signalling involves all the immune and cancer cells (Figure 2f).

The other top chemokine signalling pathways in TME were CXCL and CCL signalling and macrophages show significant secretion of these chemokines (Figure S2d). For instance, *CXCL16* is a chemoattractant for the Treg cells to the tumour site and its pro-tumourigenic functions^39^, and here we show the macrophages and dendritic cells recruits Treg, Th 1 helper CD4+ T and other CD8+ T cells (Figure 2g). Recently, the *CCL20*-*CCR6* axis has been known to promote cancer progression by enabling cancer cell migration and proliferation via CCL20 signalling also known as macrophage inflammatory protein^40^ and in CCC macrophages recruit Treg, CD8+ T and effector CD4+ T cells (Figure 2h). Interleukins play a critical role in promoting cancer progression while essential for tumour-directed immune response^41^, the interleukins prominent in the CCC TME are *IL1*, *IL2*, *IL6*, *IL16* and *IL10* (Figure S2d). IL1 signalling promotes angiogenesis^42^, and macrophages direct signalling to endothelial cells to promote angiogenesis. Furthermore, *IL1* is shown in the conversion of macrophages towards the M2 phenotype^42^, and there is evident *IL1A* signalling between the two macrophage populations in TME (Figure 2i). Another pathway is the CALCR pathway with the *ADM*-*CALCRL* communication, the macrophages and cancer-associated fibroblasts signal the endothelial cells via ADM secretion, suggesting the promotion of angiogenesis (Figure 2j). *ADM* has been indicated in the upregulation of the *VEGF* pathway promoting angiogenesis in ovarian carcinoma^43^. This shows that cellular communication in the CCC TME promotes angiogenesis and has a pro-tumourigenic microenvironment in the patients.

### Master regulators governing CCC TME

The key regulatory transcription factors (TFs) for each cell population in the CCC TME were identified using SCENIC^44^. The critical master regulators of CCC cancer cells were *HNF1B* and *SOX4* (Figure 2k, S2e). *HNF1B* is a hepatocyte-specific TF expressed in several cancers, and the reduction of *HNF1B* levels in CCC cell lines has been shown to cause apoptosis^45^. Similarly, *SOX4* is upregulated in subtypes of cancerous cells of clear cell renal carcinoma^46^. *SOX4* plays a key role in maintaining the stemness of cancer cells^47^ and is a known master regulator of EMT pathways in breast cancer^48^. M2 macrophages in CCC showed high *NFE2L3* regulon activity and *NFE2L3* expression is a poor prognosis marker in colon, pancreatic and renal cancers^49–51^. A study has also shown that loss of *NFE2L3* protects against inflammation caused by colorectal cancer by modulating TME^52^. Interestingly, exhausted T cells had higher regulon activity of *IRF4* compared to CD4 and CD8 T cells (Figure 2k). *IRF4* has been shown to drive the exhaustion of CD8+T cells in chronic infection^53^. Overall thesignalling pathways and transcriptional regulators in CCC are pro-tumourgenic.

### Progression of CCC cancer cells

**Figure 3:**
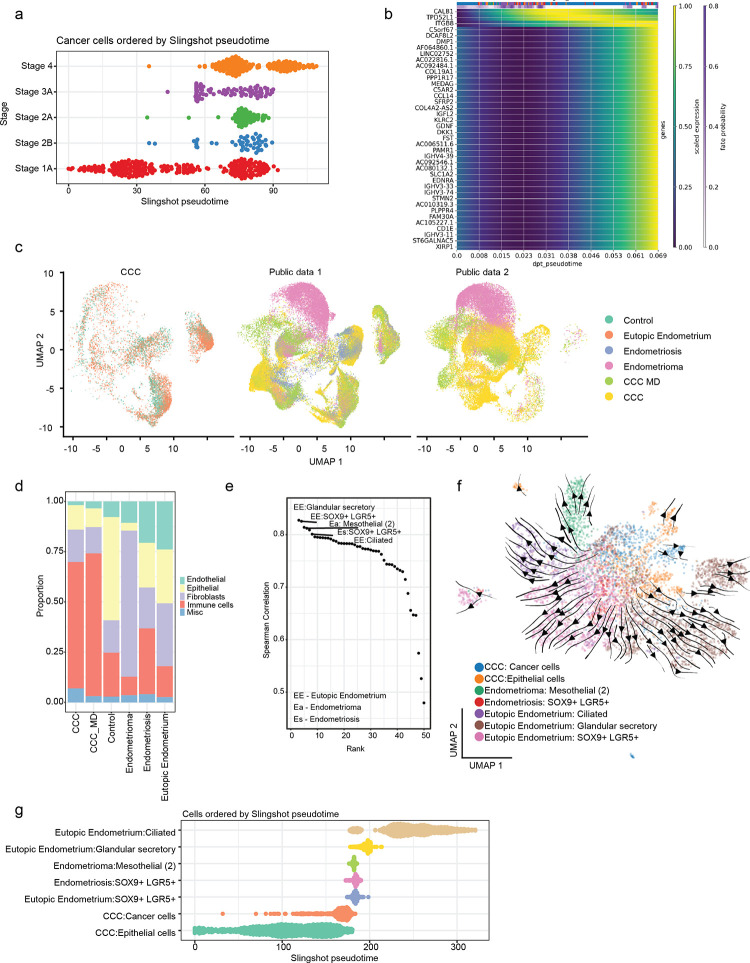
Deciphering the CCC cancer cells progression and origin of epithelial cells. a) The cancer cells are ordered by the stages of cancer based on slingshot pseudotime. b) The changes in gene expression across the diffusion pseudo-time from stage 1 to 4 cancer cells, along with the cell fate probability changes. c) UMAP visualisation of integration of CCC tumour samples in this study with healthy control, eutopic endometrium, endometrioma and endometriosis from two publicly available datasets. d) The proportion of cell groups present in each condition. e) The Spearman correlation of the transcriptional profiles between the cancer cells in CCC and the epithelial cells found in healthy controls and other endometrial conditions. The cell types with a Spearman correlation greater than 0.8 with CCC cancer cells are labelled. f) The UMAP visualisation of the CCC cancer cells and the correlated epithelial cell types. The projection lines and arrows represent the pseudotime. g) The correlated epithelial cells are ordered by slingshot pseudotime.

Next, we aimed to study the nature of CCC cancer cell progression across the four cancer stages. Interestingly, the more advanced stages of cancer stages 3 and 4 have reduced responses to chemokines and humoral immune responses based on differential gene ontology (Figure S3a). The chemokines are usually responsible for the recruitment of immune cells to the tumour site and humoral immunity comprising of B cells is important for tumour suppression. Here, the advanced stages of CCC seem to be immune-compromised. Further, cell trajectory analysis was performed to map the progression of cancer cells in CCC (Figure 3a, S3b-c). We observed that the stage 1 cancer cells are on one end of the spectrum, and metastasised stage 4 cancer cells are on the other, demonstrating the cancer cells’ overall progression. Next, the cell fate probabilities were determined to identify the gene expression changes during cancer stage progression (Figure S3c). The sequential genes upregulated during the progression from cancer stage 1 to 4 are *CALB1*, *TPD52L1* and *ITGB8* (Figure 3b). *CALB1* is an oncogene known in ovarian cancer and acts by inhibiting p53 pathway^54^. Similarly, *TPD52L1*^55^ *and ITGB8*^56^ are known to be in other cancer transitions. Additionally, in metastasised stage 4 cancer, ligands such as *GDNF* and *DKK1* are also responsible for tumour progression^57^, especially *DKK1* is known to promote tumour metastasis^58^.

### Cellular composition of various endometrial conditions

Since CCC is associated with endometriosis, we compared CCC with healthy endometrium and endometriosis-related conditions using two publicly available scRNA-seq datasets^24,25^. Firstly, we combined the single cells in the two datasets and grouped them as “control” representing healthy endometrium, “eutopic endometrium” representing the healthy endometrium of donors with endometriosis, “endometriosis” representing the tissue with endometriosis and “endometrioma” which are cystic lesions that stem from endometriosis. The integrated scRNA-seq data from this study and public datasets comprising six conditions such as control, eutopic endometrium, endometriosis, endometrioma, CCC and CCC-MD (CCC with a mixed diagnosis of endometriosis) are shown via UMAP dimension reduction projection (Figure 3c).

To understand the composition of the tissue in different endometrial conditions, the proportion of cell types present in each condition was determined (Figure 3d, Figure S3d-g). The endometrial region has an abundance of immune cells, with the healthy control containing more than 20% immune cells and 50% epithelial cells. During the endometriosis condition, we can already observe an increase in immune and endothelial cells by more than 10% and a reduction in the proportion of epithelial cells. The population of immune cells further increases to 65% in cancerous conditions like the CCC and CCC-MD, suggesting an immune infiltration. Meanwhile, the endometrioma cyst is predominantly made of fibroblasts and fewer immune cells.

### Deciphering the origin of CCC cancer cells

Endometriosis is defined by the presence of endometrial tissue in regions other than the endometrium. As women with endometriosis are more likely to get CCC, we aimed to decipher whether endometriosis is a precursor or facilitator of CCC by comparing the cancer cells with the epithelial cells found in endometrium and other endometriosis conditions. The transcriptional similarity was detected by Spearman correlation between CCC cancer cells and epithelial cells found in other conditions (Figure 3e, Table S2). The transcriptionally closest epithelial cells were glandular secretory, SOX9+ LGR5+ epithelial cells and ciliated in the eutopic endometrium of donors with endometriosis. The glandular secretory and ciliated epithelial cells are found in the luteal phase, and SOX9+ LGR5+ epithelial cells are found in the follicular phase of the menstrual cycle^25^. These cell types are transcriptional-related closely based on UMAP projection and pseudotime trajectory (Figure 3f–e). Further, gene ontology analysis also reveals the similarity between the cell types (Figure S3h). The one key signalling pathway active in cancer cells and not in the other compared to epithelial cells was the CyP signalling, known in poor tumour prognosis^38^ (Figure S4a, Figure 2f). Interestingly, it was shown that glandular secretory epithelial cells have high expression of *ARID1A* and *KRAS*^25^ and this is correlated with ovarian clear cell carcinoma samples^59^. These findings suggest that the CCC cancer cells might have originated from the epithelial cells related to the endometriosis condition such as glandular/secretory, ciliated and SOX9+ LGR5+ epithelial cells.

### Cellular communication in the microenvironment of endometrial conditions and CCC

**Figure 4:**
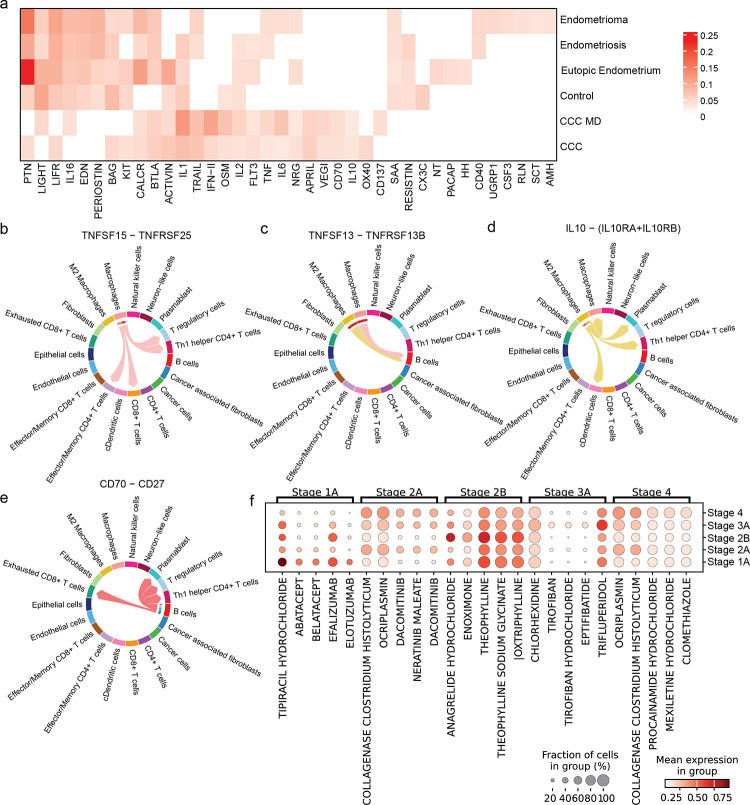
Comparison of cell-cell communication between conditions and prediction of potential drugs in CCC. a) The heatmap shows the distinct signalling pathways enriched across different conditions. b) The predicted drugs against the transcriptome profile of the TME across the stages of cancer. The chord plots show the ligand and receptor interaction among the cell types in the TME for the following selected signalling pathways in CCC for b) APRIL pathway with *TNFSF15* ligand and *TNFRSF25* receptor, c) VEGI pathway with *TNFSF13* ligand and *TNFRSF13B* receptor, d) IL10 pathway with *IL10* ligand and *IL10RA-IL10RB* receptor, e) CD70 pathway with *CD70* ligand and *CD27* receptor. The colour of the connections between cell types represents the ligands expressed from the cell type and the arrow points to the receptor of the cell types. f) The dot plot shows the predicted drugs targeting the transcriptional profile of the TME at different stages of cancer.

It is necessary to decipher the differences in cell-cell communication to understand the dynamics of different endometrial conditions and CCC. Here, we performed cell-cell communication analysis based on the transcriptional profiles of the ligands and receptors in cell types found in the microenvironment. The major signalling pathways specific to the cancer condition are VEGI, APRIL, IL10, and CD70 (Figure 4a), and these pathways mostly involve the signalling from immune cells. The macrophages recruit the T cells, including the Treg and Th1 helper cells via VEGI (TNFSF15) signalling (Figure 4b), and it has been shown that *TNFSF15* promotes macrophage polarisation toward M1 for tumour suppression^60^. On the other hand, *APRIL* (*TNFSF13*) is known to stimulate B cell growth (Figure 4c) and cancer cell growth^61^. Further, *IL10* an anti-inflammatory ligand, is secreted by M2 macrophages to Th1 helper T cells and dendritic cells (Figure 4d). Finally, dysregulation of the CD70-CD27 axis within TME has been associated with tumour progression and immunosuppression^62^, and we observed significant signalling between B cells, Tregs, and exhausted T cells (Figure 4e). The *OX40* and *CD137* show differential presence in CCC and CCC with mixed diagnosis of endometriosis. Interestingly, the CALCR and BTLA pathways are present in both endometriosis conditions and CCC but not in healthy tissue, indicating a dysregulation in the calcium pathway and immune checkpoint, respectively.

### Repurposing drugs targeting the TME

To predict the potential repurposing drugs for CCC, we aimed to predict the drugs based on the transcriptional profile of the TME. We stratified the patients based on the stages of cancer and used drug2cell^63^ to predict the drugs that could potentially target the TME (Figure 4f, S4b-c). The most significant drug predicted for Stage 1 TME is Tipiracil hydrochloride, which is a thymidine phosphorylase inhibitor used for the treatment of colorectal cancer^64^. The remaining top predicted drugs for Stage 1 were Abatacept and Belatacept targeting the T cells, Efalizumab targeting lymphocytes and Elotuzumab, which has an anti-tumour function by enhancing activation of natural killer cells based on the DrugBank database^65^. Some of the top predicted drugs for Stage 2 were Dacomitinib and Neratinib, and these drugs are used to treat non-small cell lung cancer and breast cancer, respectively. The drugs predicted for higher stages of CCC were drugs for preventing cardiovascular or asthma, suggesting the effects of medication the patients are already taking. Overall, the scRNA-seq profiling of CCC TME enables a detailed understanding of cell-cell communication and the possibility of identifying drugs for repurposing and working towards helping in personalised medicine.

## Discussion

This is the first study to profile single cells from clear cell carcinoma TME during different stages of cancer progression. We decipher the cellular communication in the TME and compare it with other endometrial-related conditions. We further show the potential of scRNA-seq to predict drugs for treatment. However, this study has some limitations, such as a smaller sample size and here we aim to use this as a discovery cohort to understand CCC progression. Another limitation is profiling was done in a single modality, therefore, as an extension, multimodal sequencing can be applied to comprehend CCC TME dynamics such as chromatin accessibility^66^, histone profiling^67^ and RNA modification^68^.

Across all donors, *MIF* and *SPP1* signalling was prominent in cancer cells while *SPP1* signalling was also active in M2 macrophages. The potential recipients of *SPP1* signalling included immune cells infiltrating tumours, cancer-associated fibroblasts, and regulatory T cells. Interestingly, *SPP1* signalling was high in CCC compared to endometrial conditions. A recent pan-cancer analysis using public data from TCGA showed that *SPP1* is overexpressed in most cancers^69^. *SPP1* has been shown to recruit macrophages to turn into tumour-associated macrophages in gliomas^70^. Anti-*SPP1* antibodies have been shown to suppress colon tumour growth in vivo^71^. *SPP1* inhibitors like parecoxib, brefelamide and simvastatin could be used as adjuvants in chemotherapy for the treatment of CCC.

Examination of the CCC TME revealed activation of several signalling pathways (HGF, ANGPTL4, HBEGF, and FGF) that promote tumour growth and angiogenesis. M2 macrophages displayed prominent *PPIA*-*BSG* signalling, previously linked to poor prognosis in various cancers. Infiltrating immune cells showed active *CXCL16* and CCL20 signalling, which aid cancer progression. Furthermore, TME recruitment of interleukins (*IL1*, *IL2*, *IL6*, *IL16*, and *IL10*) further promotes angiogenesis. Finally, *ADM* signalling, which upregulates VEGF signalling to promote blood vessel growth, was active in macrophages, CAFs, and endothelial cells.

Gene regulatory network analysis revealed distinct transcription factor activity in different cell types within the CCC tumour microenvironment. Exhausted T cells displayed the strongest regulon activity of *IRF4*, a transcription factor known to drive T-cell exhaustion during chronic inflammation. Blocking *IRF4* could be a potential therapeutic strategy to reinvigorate exhausted T cells and enhance anti-tumor immunity. Cancer cells, on the other hand, showed high expression of *HNF1B*, contributing to maintaining stemness in cancer cells, a characteristic essential for tumour growth. This finding suggests that *HNF1B* is another potential target for therapeutic intervention in CCC.

To understand the unique molecular signature of CCC, we compared it with endometriosis and healthy endometrium. As prior research indicates, women with endometriosis have a threefold increased risk of developing CCC. This comparison aimed to identify potential causative links by analysing cell-cell communication patterns within both conditions. We determined pathways specific to CCC, such as OX40 and CD137, by excluding signalling pathways common to endometriosis and healthy endometrium.

Interestingly, CCC cancer cells displayed transcriptional similarity to glandular secretory, ciliated, and SOX9+ LGR5+ epithelial found in endometriosis conditions. Furthermore, ARID1A, a well-established oncogene in CCC, is highly expressed in these same endometriosis cells^25^. This is particularly noteworthy as mutations in *ARID1A* are found in 57% of ovarian CCC cases^72^. Based on this shared expression of *ARID1A* and the observed transcriptional similarities, we postulate that ovarian CCC cells may originate from these specific epithelial cells present in the endometriosis condition.

Finally, we aim to find druggable targets during different stages of cancer progression. Tipiracil hydrochloride, which has been used for the treatment of colorectal cancer, was shown to be drug-specific for stage 1 of CCC. For Stage 2, Dacomitinib and Neratinib were predicted to counter CCC. These drugs are used to treat non-small cell lung cancer and breast cancer, respectively. Established based on this cohort, we show how we could determine potential candidates using scRNA-seq for patient-specific drug interventions in ovarian CCC.

## Methods

### Sample acquisition

This study’s approval was obtained from the SingHealth Centralized Institutional Review Board, encompassing the National Cancer Centre Singapore, Singapore General Hospital, and Kandang Kerbau Women’s and Children’s Hospital (CIRB 2015/2595). Tissue samples from ovarian cancer patients with informed consent were obtained ex vivo and dissected freshly by the pathologist. Samples were collected in RPMI 1640 medium and delivered on ice to the laboratory within 1 hour of tissue collection.

### Processing the sample

Tissue samples of ovarian tumours were cut into ~1–2 mm^3^ pieces using sterile scalpels and digested with collagenase type IV (STEMCELL Technologies) for 2 hours at 37°C on a rotator. Cell suspensions were filtered through a 70 mm cell strainer and centrifuged, and red blood cells (RBC) in the cell pellets were removed through RBC lysis. Cells were washed with PBS and snap frozen with liquid nitrogen and stored at −80°C until ready for single-cell analysis experimental use. The sample was dissociated, and the single-cell suspension was prepared by trypsin treatment for 2–3 hours at 37 C. The single-cell suspension was processed for single-cell analysis using 10× genomics single-cell RNAseq protocol. The single-cell library was sequenced using the Hiseq 4000 Illumina sequencer.

### scRNA-seq data processing

Cellranger v7.1 was used to map the 10X scRNA-seq data to GRCh38 v32 human reference genomes. The resulting count data was analysed using Seurat v4.3^73^. The cells were filtered out if the cells contained less than 500 genes or greater than 25% mitochondria reads. The cells with UMI counts less than 0.01% quantile and greater than 95% quantile across all cells were removed. The genes were filtered out if expressed in less than 10 cells. The genes with log10 of average UMI count across all cells greater than −2.5, genes with at least 2 UMIs and genes detected in at least 10 cells were used for downstream analysis. DoubletFinder v2.0.3^74^ was used to identify and remove doublets.

### Cell type annotation

Next, to annotate the cell types, we used scATOMIC^26^ a machine-learning cell annotation method trained using public pan-cancer scRNA-seq data. We additionally manually curated the annotation using well-known marker genes for each cell type.

### Integration of across samples and studies

The gene count for each tumour sample was normalised using SCTransform^75^ and dimension reduction was performed using PCA and UMAP. The seven samples were integrated using the Pearson residuals integration method from Seurat SCTransform integration, resulting in 13,543 cells.

We integrated publicly available endometriosis datasets^24,25^ with 93,766 and 65,903 single cells to compare with our CCC data using the Pearson residuals integration method from Seurat SCTransform integration.

### Differential single-cell RNA-seq analysis

Pairwise differential expression analysis was performed using MAST v1.12.0^76^ on genes expressed in at least 0.25 fractions of the cells. Genes are differentially expressed if a minimum log fold-change threshold is greater than 0.25 and a p-value is less than 0.05.

### Gene set enrichment analysis

The gene set enrichment analysis was performed in clusterProfiler^77^ on the identified differentially expressed genes.

### Trajectory analysis

Using Spearman correlation, similar epithelial cell types across all endometrial conditions and CCC were identified. Then, pseudo-time trajectory analysis was carried out using Slingshot^78,79^ on the PCA projection and CellRank^78^ on the UMAP projection. Diffusion pseudotime was calculated to determine the genes that change across stages of cancer.

### Cell-cell communication analysis

The changes in overall signalling pathway communication between different cell types present within the CCC TME and other endometrial conditions were computed and visualised as dot plots and chord plots using CellChat^80^.

### SNPs detection

The SNPs were detected in the scRNA-seq data using scAllele^81^. The identified SNPs were analysed and visualised using Maftools^82^.

### Drug prediction

Drugs targeting the TME in CCC at different stages of cancer and different cell types were computed using drug2cell^63^ based on the enrichment of drugs for the transcriptional signatures.

## Data Availability

The scRNA-seq data was visualised using an interactive website developed using ShinyCell^83^. The scRNA-seq data generated in this study will be made available on the GEO database. The website files will be made available on GitHub.

## References

[1] Sung H. Global Cancer Statistics 2020: GLOBOCAN Estimates of Incidence and Mortality Worldwide for 36 Cancers in 185 Countries. CA Cancer J. Clin. 71, 209–249 (2021). 33538338 10.3322/caac.21660

[2] Siegel R. L., Miller K. D. & Jemal A. Cancer statistics, 2020. CA Cancer J. Clin. 70, 7–30 (2020). 31912902 10.3322/caac.21590

[3] Ferlay J. Estimating the global cancer incidence and mortality in 2018: GLOBOCAN sources and methods. Int. J. Cancer 144, 1941–1953 (2019). 30350310 10.1002/ijc.31937

[4] Bray F. Global cancer statistics 2018: GLOBOCAN estimates of incidence and mortality worldwide for 36 cancers in 185 countries. CA Cancer J. Clin. 68, 394–424 (2018). 30207593 10.3322/caac.21492

[5] Oliver K. E. An evaluation of progression free survival and overall survival of ovarian cancer patients with clear cell carcinoma versus serous carcinoma treated with platinum therapy: An NRG Oncology/Gynecologic Oncology Group experience. Gynecol. Oncol. 147, 243–249 (2017). 28807367 10.1016/j.ygyno.2017.08.004 PMC5697899

[6] Ku F.-C. Clear cell carcinomas of the ovary have poorer outcomes compared with serous carcinomas: Results from a single-center Taiwanese study. J. Formos. Med. Assoc. 117, 117–125 (2018). 28389144 10.1016/j.jfma.2017.03.007

[7] Jacoby V. L., Fujimoto V. Y., Giudice L. C., Kuppermann M. & Washington A. E. Racial and ethnic disparities in benign gynecologic conditions and associated surgeries. Am. J. Obstet. Gynecol. 202, 514–521 (2010). 20430357 10.1016/j.ajog.2010.02.039 PMC4625911

[8] Krämer P. Endometrial Cancer Molecular Risk Stratification is Equally Prognostic for Endometrioid Ovarian Carcinoma. Clin. Cancer Res. 26, 5400–5410 (2020). 32737030 10.1158/1078-0432.CCR-20-1268

[9] Miyazawa K. Incidence of endometriosis among Japanese women. Obstet. Gynecol. 48, 407–409 (1976). 967375

[10] Arumugam K. & Lim J. M. Menstrual characteristics associated with endometriosis. Br. J. Obstet. Gynaecol. 104, 948–950 (1997). 9255089 10.1111/j.1471-0528.1997.tb14357.x

[11] DePriest P. D. Endometrioid carcinoma of the ovary and endometriosis: the association in postmenopausal women. Gynecol. Oncol. 47, 71–75 (1992). 1427405 10.1016/0090-8258(92)90079-x

[12] Heaps J. M., Nieberg R. K. & Berek J. S. Malignant neoplasms arising in endometriosis. Obstet. Gynecol. 75, 1023–1028 (1990). 2188180

[13] Jimbo H. Prevalence of ovarian endometriosis in epithelial ovarian cancer. Int. J. Gynaecol. Obstet. 59, 245–250 (1997). 9486515 10.1016/s0020-7292(97)00238-5

[14] Oral E. Prevalence of endometriosis in malignant epithelial ovary tumours. Eur. J. Obstet. Gynecol. Reprod. Biol. 109, 97–101 (2003). 12818453 10.1016/s0301-2115(03)00047-2

[15] Brinton L. A., Gridley G., Persson I., Baron J. & Bergqvist A. Cancer risk after a hospital discharge diagnosis of endometriosis. Am. J. Obstet. Gynecol. 176, 572–579 (1997). 9077609 10.1016/s0002-9378(97)70550-7

[16] Pearce C. L. Association between endometriosis and risk of histological subtypes of ovarian cancer: a pooled analysis of case-control studies. Lancet Oncol. 13, 385–394 (2012). 22361336 10.1016/S1470-2045(11)70404-1 PMC3664011

[17] Zondervan K. T. Endometriosis. Nat Rev Dis Primers 4, 9 (2018). 30026507 10.1038/s41572-018-0008-5

[18] Sampson J. A. The development of the implantation theory for the origin of peritoneal endometriosis. Am. J. Obstet. Gynecol. 40, 549–557 (1940).

[19] Anglesio Michael S. Cancer-Associated Mutations in Endometriosis without Cancer. N. Engl. J. Med. 376, 1835–1848. 10.1056/NEJMoa1614814 PMC5555376 28489996

[20] Yamamoto S. PIK3CA mutation is an early event in the development of endometriosis-associated ovarian clear cell adenocarcinoma. J. Pathol. 225, 189–194 (2011). 21735444 10.1002/path.2940

[21] Tan T. Z. Analysis of gene expression signatures identifies prognostic and functionally distinct ovarian clear cell carcinoma subtypes. EBioMedicine 50, 203–210 (2019). 31761620 10.1016/j.ebiom.2019.11.017 PMC6921362

[22] Gautam P. Multi-species single-cell transcriptomic analysis of ocular compartment regulons. Nat. Commun. 12, 5675 (2021). 34584087 10.1038/s41467-021-25968-8 PMC8478974

[23] Ng M. S. F. Deterministic reprogramming of neutrophils within tumors. Science 383, eadf6493 (2024). 38207030 10.1126/science.adf6493 PMC11087151

[24] Tan Y. Single-cell analysis of endometriosis reveals a coordinated transcriptional programme driving immunotolerance and angiogenesis across eutopic and ectopic tissues. Nat. Cell Biol. 24, 1306–1318 (2022). 35864314 10.1038/s41556-022-00961-5 PMC9901845

[25] Fonseca M. A. S. Single-cell transcriptomic analysis of endometriosis. Nat. Genet. 55, 255–267 (2023). 36624343 10.1038/s41588-022-01254-1 PMC10950360

[26] Nofech-Mozes I., Soave D., Awadalla P. & Abelson S. Pan-cancer classification of single cells in the tumour microenvironment. Nat. Commun. 14, 1615 (2023). 36959212 10.1038/s41467-023-37353-8 PMC10036554

[27] Zeng J. CancerSCEM: a database of single-cell expression map across various human cancers. Nucleic Acids Res. 50, D1147–D1155 (2022). 34643725 10.1093/nar/gkab905 PMC8728207

[28] Zuccolo J. Expression of MS4A and TMEM176 Genes in Human B Lymphocytes. Front. Immunol. 4, 195 (2013). 23874341 10.3389/fimmu.2013.00195 PMC3711070

[29] Syn N. L., Teng M. W. L., Mok T. S. K. & Soo R. A. De-novo and acquired resistance to immune checkpoint targeting. Lancet Oncol. 18, e731–e741 (2017). 29208439 10.1016/S1470-2045(17)30607-1

[30] Rudensky A. Y. Regulatory T cells and Foxp3. Immunol. Rev. 241, 260–268 (2011). 21488902 10.1111/j.1600-065X.2011.01018.x PMC3077798

[31] Wang X. New insights from the single-cell level: Tumor associated macrophages heterogeneity and personalized therapy. Biomed. Pharmacother. 153, 113343 (2022). 35785706 10.1016/j.biopha.2022.113343

[32] Yuan Q. CAFrgDB: a database for cancer-associated fibroblasts related genes and their functions in cancer. Cancer Gene Ther. 30, 917–925 (2023). 36922546 10.1038/s41417-023-00603-4 PMC10281871

[33] Chandler R. L. Coexistent ARID1A-PIK3CA mutations promote ovarian clear-cell tumorigenesis through pro-tumorigenic inflammatory cytokine signalling. Nat. Commun. 6, 6118 (2015). 25625625 10.1038/ncomms7118 PMC4308813

[34] Mora Barthelmess R., Stijlemans B. & Van Ginderachter J. A. Hallmarks of Cancer Affected by the MIF Cytokine Family. Cancers 15, (2023). 10.3390/cancers15020395 PMC9856758 36672343

[35] Gao W. SPP1 is a prognostic related biomarker and correlated with tumor-infiltrating immune cells in ovarian cancer. BMC Cancer 22, 1367 (2022). 36585688 10.1186/s12885-022-10485-8 PMC9805166

[36] Xu Y. & Shen K. Identification of potential key genes associated with ovarian clear cell carcinoma. Cancer Manag. Res. 10, 5461–5470 (2018). 30519094 10.2147/CMAR.S187156 PMC6234989

[37] Roles of MET in human cancer. Clin. Chim. Acta 525, 69–83 (2022). 34951962 10.1016/j.cca.2021.12.017

[38] Han J. M. & Jung H. J. Cyclophilin A/CD147 Interaction: A Promising Target for Anticancer Therapy. Int. J. Mol. Sci. 23, (2022). 10.3390/ijms23169341 PMC9408992 36012604

[39] Korbecki J. The Role of CXCL16 in the Pathogenesis of Cancer and Other Diseases. Int. J. Mol. Sci. 22, (2021). 10.3390/ijms22073490 PMC8036711 33800554

[40] Kadomoto S., Izumi K. & Mizokami A. The CCL20-CCR6 Axis in Cancer Progression. Int. J. Mol. Sci. 21, (2020). 10.3390/ijms21155186 PMC7432448 32707869

[41] Briukhovetska D. Interleukins in cancer: from biology to therapy. Nat. Rev. Cancer 21, 481–499 (2021). 34083781 10.1038/s41568-021-00363-z PMC8173513

[42] Watari K. Tumor-derived interleukin-1 promotes lymphangiogenesis and lymph node metastasis through M2-type macrophages. PLoS One 9, e99568 (2014). 24924428 10.1371/journal.pone.0099568 PMC4055709

[43] Chen Q., Chen P., Pang X., Hu Y. & Zhang Y. Adrenomedullin Up-regulates the Expression of Vascular Endothelial Growth Factor in Epithelial Ovarian Carcinoma Cells via JNK/AP-1 Pathway. Int. J. Gynecol. Cancer 25, 953–960 (2015). 26098087 10.1097/IGC.0000000000000465 PMC4485736

[44] Aibar S. SCENIC: single-cell regulatory network inference and clustering. Nat. Methods 14, 1083–1086 (2017). 28991892 10.1038/nmeth.4463 PMC5937676

[45] Tsuchiya A. Expression profiling in ovarian clear cell carcinoma: identification of hepatocyte nuclear factor-1 beta as a molecular marker and a possible molecular target for therapy of ovarian clear cell carcinoma. Am. J. Pathol. 163, 2503–2512 (2003). 14633622 10.1016/s0002-9440(10)63605-x PMC1892387

[46] Pan X.-W. Molecular subtyping and characterization of clear cell renal cell carcinoma by tumor differentiation trajectories. iScience 26, 108370 (2023). 38034348 10.1016/j.isci.2023.108370 PMC10682269

[47] Liu J. SOX4 maintains the stemness of cancer cells via transcriptionally enhancing HDAC1 revealed by comparative proteomics study. Cell Biosci. 11, 23 (2021). 33482915 10.1186/s13578-021-00539-y PMC7821488

[48] Parvani J. G. & Schiemann W. P. Sox4, EMT programs, and the metastatic progression of breast cancers: mastering the masters of EMT. Breast Cancer Res. 15, R72 (2013). 23981787 10.1186/bcr3466 PMC3979076

[49] Zhang Q. NFE2L3 as a Potential Functional Gene Regulating Immune Microenvironment in Human Kidney Cancer. Biomed Res. Int. 2022, 9085186 (2022). 36337840 10.1155/2022/9085186 PMC9629961

[50] Bury M. NFE2L3 Controls Colon Cancer Cell Growth through Regulation of DUX4, a CDK1 Inhibitor. Cell Rep. 29, 1469–1481.e9 (2019). 31693889 10.1016/j.celrep.2019.09.087

[51] Alam M. M. Multiomics analysis of altered NRF3 expression reveals poor prognosis in cancer. Informatics in Medicine Unlocked 29, 100892 (2022).

[52] Saliba J. Loss of NFE2L3 protects against inflammation-induced colorectal cancer through modulation of the tumor microenvironment. Oncogene 41, 1563–1575 (2022). 35091681 10.1038/s41388-022-02192-2 PMC8913363

[53] Man K. Transcription Factor IRF4 Promotes CD8+ T Cell Exhaustion and Limits the Development of Memory-like T Cells during Chronic Infection. Immunity 47, 1129–1141.e5 (2017). 29246443 10.1016/j.immuni.2017.11.021

[54] Cao L.-Q., Wang Y.-N., Liang M. & Pan M.-Z. CALB1 enhances the interaction between p53 and MDM2, and inhibits the senescence of ovarian cancer cells. Mol. Med. Rep. 19, 5097–5104 (2019). 31059057 10.3892/mmr.2019.10212 PMC6522887

[55] Boutros R. & Byrne J. A. D53 (TPD52L1) is a cell cycle-regulated protein maximally expressed at the G2-M transition in breast cancer cells. Exp. Cell Res. 310, 152–165 (2005). 16112108 10.1016/j.yexcr.2005.07.009

[56] Liu Y. Paradoxical role of β8 integrin on angiogenesis and vasculogenic mimicry in glioblastoma. Cell Death Dis. 13, 536 (2022). 35676251 10.1038/s41419-022-04959-7 PMC9177864

[57] Fielder G. C. The GDNF Family: A Role in Cancer? Neoplasia 20, 99–117 (2018). 29245123 10.1016/j.neo.2017.10.010 PMC5730419

[58] Zhu G. Expression and Role of Dickkopf-1 (Dkk1) in Tumors: From the Cells to the Patients. Cancer Manag. Res. 13, 659–675 (2021). 33536782 10.2147/CMAR.S275172 PMC7847771

[59] Tate J. G. COSMIC: the Catalogue Of Somatic Mutations In Cancer. Nucleic Acids Res. 47, D941–D947 (2019). 30371878 10.1093/nar/gky1015 PMC6323903

[60] Zhao C.-C. TNFSF15 facilitates differentiation and polarization of macrophages toward M1 phenotype to inhibit tumor growth. Oncoimmunology 11, 2032918 (2022). 35127254 10.1080/2162402X.2022.2032918 PMC8812784

[61] Nowacka K. H. & Jabłońska E. Role of the APRIL molecule in solid tumors. Cytokine Growth Factor Rev. 61, 38–44 (2021). 34446365 10.1016/j.cytogfr.2021.08.001

[62] Flieswasser T. The CD70-CD27 axis in oncology: the new kids on the block. J. Exp. Clin. Cancer Res. 41, 12 (2022). 34991665 10.1186/s13046-021-02215-y PMC8734249

[63] Kanemaru K. Spatially resolved multiomics of human cardiac niches. Nature 619, 801–810 (2023). 37438528 10.1038/s41586-023-06311-1 PMC10371870

[64] Stavraka C. Trifluridine/Tipiracil in Metastatic Colorectal Cancer: A UK Multicenter Real-world Analysis on Efficacy, Safety, Predictive and Prognostic Factors. Clin. Colorectal Cancer 20, 342–349 (2021). 34696965 10.1016/j.clcc.2021.09.009

[65] Wishart D. S. DrugBank: a knowledgebase for drugs, drug actions and drug targets. Nucleic Acids Res. 36, D901–6 (2008). 18048412 10.1093/nar/gkm958 PMC2238889

[66] Xing Q. R. Parallel bimodal single-cell sequencing of transcriptome and chromatin accessibility. Genome Res. 30, 1027–1039 (2020). 32699019 10.1101/gr.257840.119 PMC7397874

[67] Warrier T. SETDB1 acts as a topological accessory to Cohesin via an H3K9me3-independent, genomic shunt for regulating cell fates. Nucleic Acids Res. 50, 7326–7349 (2022). 35776115 10.1093/nar/gkac531 PMC9303280

[68] Hamashima K. Single-nucleus multiomic mapping of mA methylomes and transcriptomes in native populations of cells with sn-m6A-CT. Mol. Cell (2023) doi:10.1016/j.molcel.2023.08.010. PMC10895704 37657444

[69] Liu Y. A pan-cancer analysis of the oncogenic role of secreted phosphoprotein 1 (SPP1) in human cancers. Ann Transl Med 10, 279 (2022). 35433956 10.21037/atm-22-829 PMC9011290

[70] Wei J. Osteopontin mediates glioblastoma-associated macrophage infiltration and is a potential therapeutic target. J. Clin. Invest. 129, 137–149 (2019). 30307407 10.1172/JCI121266 PMC6307970

[71] Klement J. D. Osteopontin Blockade Immunotherapy Increases Cytotoxic T Lymphocyte Lytic Activity and Suppresses Colon Tumor Progression. Cancers 13, (2021). 10.3390/cancers13051006 PMC7957528 33670921

[72] Jones S. Frequent mutations of chromatin remodeling gene ARID1A in ovarian clear cell carcinoma. Science 330, 228–231 (2010). 20826764 10.1126/science.1196333 PMC3076894

[73] Hao Y. Integrated analysis of multimodal single-cell data. Cell 184, 3573–3587.e29 (2021). 34062119 10.1016/j.cell.2021.04.048 PMC8238499

[74] McGinnis C. S., Murrow L. M. & Gartner Z. J. DoubletFinder: Doublet Detection in Single-Cell RNA Sequencing Data Using Artificial Nearest Neighbors. Cell Syst 8, 329–337.e4 (2019). 30954475 10.1016/j.cels.2019.03.003 PMC6853612

[75] Hafemeister C. & Satija R. Normalization and variance stabilization of single-cell RNA-seq data using regularized negative binomial regression. Genome Biol. 20, 296 (2019). 31870423 10.1186/s13059-019-1874-1 PMC6927181

[76] Finak G. MAST: a flexible statistical framework for assessing transcriptional changes and characterizing heterogeneity in single-cell RNA sequencing data. Genome Biol. 16, 278 (2015). 26653891 10.1186/s13059-015-0844-5 PMC4676162

[77] Wu T. clusterProfiler 4.0: A universal enrichment tool for interpreting omics data. Innovation (Camb) 2, 100141 (2021). 34557778 10.1016/j.xinn.2021.100141 PMC8454663

[78] Lange M. CellRank for directed single-cell fate mapping. Nat. Methods 19, 159–170 (2022). 35027767 10.1038/s41592-021-01346-6 PMC8828480

[79] Street K. Slingshot: cell lineage and pseudotime inference for single-cell transcriptomics. BMC Genomics 19, 477 (2018). 29914354 10.1186/s12864-018-4772-0 PMC6007078

[80] Jin S. Inference and analysis of cell-cell communication using CellChat. Nat. Commun. 12, 1088 (2021). 33597522 10.1038/s41467-021-21246-9 PMC7889871

[81] Quinones-Valdez G., Fu T., Chan T. W. & Xiao X. scAllele: A versatile tool for the detection and analysis of variants in scRNA-seq. Sci Adv 8, eabn6398 (2022). 36054357 10.1126/sciadv.abn6398 PMC11636672

[82] Mayakonda A., Lin D.-C., Assenov Y., Plass C. & Koeffler H. P. Maftools: efficient and comprehensive analysis of somatic variants in cancer. Genome Res. 28, 1747–1756 (2018). 30341162 10.1101/gr.239244.118 PMC6211645

[83] Ouyang J. F., Kamaraj U. S., Cao E. Y. & Rackham O. J. L. ShinyCell: Simple and sharable visualisation of single-cell gene expression data. Bioinformatics (2021) doi:10.1093/bioinformatics/btab209. 33774659

